# 16S rRNA gene sequencing reveals the correlation between the gut microbiota and the susceptibility to pathological scars

**DOI:** 10.3389/fmicb.2023.1215884

**Published:** 2023-06-20

**Authors:** Ming Li, Minghao Li, Yingting Dai, Dang Li, Han Yu, Jian Liu, Hangqi Gao, Yi Zhong, Mingquan Huang, Jing Lin, Yide Xie, Zhihui Guo, Xiaosong Chen

**Affiliations:** ^1^Department of Plastic Surgery and Regenerative Medicine, Fujian Medical University Union Hospital, Fuzhou, China; ^2^Department of Plastic Surgery and Regenerative Medicine Institute, Fujian Medical University, Fuzhou, China; ^3^Engineering Research Center of Tissue and Organ Regeneration, Fujian Province University, Fuzhou, China; ^4^Nursing Department of Fujian Medical University Union Hospital, Fuzhou, China; ^5^Department of Dermatology, Pingtan Comprehensive Experimental Area Hospital, Fuzhou, China; ^6^Fuzhou MineButy Clinics, Fuzhou, China

**Keywords:** pathological scars, susceptibility, gut microbiota, 16S rRNA sequencing, dysbiosis

## Abstract

The gut microbiome profile in patients with pathological scars remains rarely known, especially those patients who are susceptible to pathological scars. Previous studies demonstrated that gut microbial dysbiosis can promote the development of a series of diseases via the interaction between gut microbiota and host. The current study aimed to explore the gut microbiota of patients who are prone to suffer from pathological scars. 35 patients with pathological scars (PS group) and 40 patients with normal scars (NS group) were recruited for collection of fecal samples to sequence the 16S ribosomal RNA (16S rRNA) V3-V4 region of gut microbiota. Alpha diversity of gut microbiota showed a significant difference between NS group and PS group, and beta diversity indicated that the composition of gut microbiota in NS and PS participants was different, which implied that dysbiosis exhibits in patients who are susceptible to pathological scars. Based on phylum, genus, species levels, we demonstrated that the changing in some gut microbiota (*Firmicutes; Bacteroides; Escherichia coli*, etc.) may contribute to the occurrence or development of pathological scars. Moreover, the interaction network of gut microbiota in NS and PS group clearly revealed the different interaction model of each group. Our study has preliminary confirmed that dysbiosis exhibits in patients who are susceptible to pathological scars, and provide a new insight regarding the role of the gut microbiome in PS development and progression.

## Introduction

Human body harbors trillions of microbial cells, which play a key role to our human life. Moreover, the highest density of these microbial cells are found in the intestinal compartment, and these microbial cells form a complex microbial community in the intestine known as gut microbiota (Lozupone et al., 2012). Numerous studies have indicated that the gut microbiota communicate with multiple distant organs through a variety of signal transduction pathways, and they are closely related to many diseases in the human body, including Alzheimer’s disease, hypertension, colon cancer (Li et al., 2017; Zheng et al., 2020; D’Argenio et al., 2022). In addition, it was reported that DNA originating from gut microbes has been found in the bloodstream of patients who are experiencing active psoriasis (Ramírez-Boscá et al., 2015). In recent years, a lot of studies have raised a concept of the gut-skin axis aiming to discover the relationship between gut microbiota and skin (Salem et al., 2018; Fang et al., 2022; Mahmud et al., 2022), previous studies revealed that there may be a positive feedback loop between dysbiosis of intestinal microbiota related to *Faecalibacterium prausnitzii* and disruption of the epithelial barrier caused by uncontrolled inflammation in the epithelium (Song et al., 2016). It was found that oral administration of *Lactobacillus plantarum HY7714* prevented ultraviolet-induced photoaging in mice by inhibiting MMP-1 expression in dermal fibroblasts (Kim et al., 2014). Moreover, De Pessemier et al. (2021) found that the gut–skin axis links the gut microbiota to skin diseases via the metabolites, gut barrier and inflammatory mediators.

Skin wound healing, a complicated pathophysiological process, is generally divided into three stages including inflammation, proliferation and reshaping phases (Martin, 1997). The formation of scar is caused by excess extracellular matrix (ECM) deposition in the place of the normal dermal tissue in the process of skin repair (Jackson et al., 2012). Pathological scars, which mainly refer to keloid and hypertrophic scars, are dermal connective-tissue disorders after dermal injury caused by inflammatory response and speed healing, and it may affect patients both esthetically and psychosocially (Lau et al., 2009; Huang et al., 2014). In addition, the treatment of pathological scars presents a significant burden for patients and has always been bothering doctors for a long term, especially those patients with multiple pathological scars (Avram et al., 2009; Pai and Cummings, 2011; Jfri et al., 2015; Ogawa, 2022). Pathological scars can be influenced by numerous local, systemic, and genetic factors that affect their characteristics and quantity (Avram et al., 2009). Most prior studies are focusing on the local lesions rather than the systemic factors in regard to scars, especially those affected by gut microbiota. Therefore, in current study, we decided to found out the relationship between gut microbiota and pathological scars.

In this study, regarding the patients who are susceptible to pathological scars, we present a pioneer work to investigate the generalizable pathological scar-associated microbial signatures and examine the relationship between the pathological scars and gut microbiota by 16S rRNA gene sequencing technology. Hence, the community composition and distribution were characterized to provide an experimental basis for future studies aimed at improving the prevention and treatment of pathological scars.

## Materials and methods

### Participants

In the current study, 35 patients with pathological scars (≥3 lesions throughout the body) were recruited, and 40 patients with normal scars were distributed to the control group. Participants met the following criteria were enrolled: for pathological scar (PS) group: developing lesions in the past year; with pathological scar-related symptoms such as itching, pain, etc; scar recurrence after a series of therapy resection, such as resection, local radiotherapy, drug injection, etc; for normal scar (NS) group: scar formation within 2 years after injury or surgery. Subjects who met any of the following criteria were excluded: taking antibiotics/microecological preparation/immune modulators/hormonal drugs/traditional Chinese medicine in the past month; with endocrine system disease/Inflammatory bowel disease/frequent diarrhea; digestive system surgical procedures within 3 years; with hemodialysis/cleansing enema or oral taking bowel cleansing agent within 2 weeks. This study was approved by the Ethics Committee of the Fujian Medical University Union hospital (No. 2021KJCX020), and all participants provided written informed consent.

### Collection of fecal samples and DNA extraction

In all participants, the fecal samples were collected into stool specimen collection tubes containing DNA stabilizer, immediately afterward, they were flash-frozen on dry ice and stored at a temperature of −80°C until analysis. The genomic DNA of microbes was extracted from fecal samples using the E.Z.N.A.^®^ soil DNA Kit (Omega Bio-tek, Norcross, GA, U.S.) following the manufacturer’s instructions. The quality and concentration of DNA were determined using 1.0% agarose gel electrophoresis and the NanoDrop^®^ ND-2000 spectrophotometer (Thermo Scientific Inc., USA), then the DNA samples were stored at a temperature of −80°C for further use.

### 16S rRNA amplicon sequencing

V3-V4, the hypervariable region of the bacterial 16S rRNA gene, were amplified with primer pairs 338F (5′-ACTC CTACGGGAGGCAGCAG-3′) and 806R(5′-GGACTACHVGGGT WTCTAAT-3′) (Liu et al., 2016) using an ABI GeneAmp^®^ 9700 PCR thermocycler (ABI, CA, USA). The PCR reaction mixture contained 4 μL 5 × Fast Pfu buffer, 2 μL 2.5 mM dNTPs, 0.8 μL forward primer (5 μM), 0.8 μL reverse primer (5 μM), 0.4 μL Fast Pfu polymerase, 0.2 μL BSA, 10 ng of template DNA, and ddH2O was added to reach a final volume of 20 μL. The PCR amplification protocol was as follows: initial denaturation at 95°C for 3 min, followed by 30 cycles of denaturing at 95°C for 30 s, annealing at 55°C for 30 s and extension at 72°C for 45 s, and single extension at 72°C for 10 min, and end at 10°C, and all samples underwent amplification in triplicate. For all samples, the PCR product was extracted from 2% agarose gel, and purified by the AxyPrep DNA Gel Extraction Kit (Axygen Biosciences, Union City, CA, USA), following the manufacturer’s instructions. The purified product was quantified using the Quantus™ Fluorometer (Promega, USA). Subsequently, the purified amplicons were combined in equimolar amounts and subjected to paired-end sequencing using an Illumina NovaSeq PE250 platform (Illumina, San Diego, USA), following the standard protocols provided by Majorbio Bio-Pharm Technology Co., Ltd. (Shanghai, China).

### Microbiome analysis and statistical analysis

The initial demultiplexing of the raw FASTQ files was performed using a custom Perl script, followed by quality filtering using fastp version 0.19.6 (Chen et al., 2018), and subsequently merging using FLASH version 1.2.7 (Magoč and Salzberg, 2011). The sequences underwent filtration and clustering to form operational taxonomic units (OTUs) using UPARSE 7.1 at a 97% similarity threshold. The taxonomy of OTU was analyzed by QIIME (Version 1.9.1) against the 16S rRNA database (Silva V138), with a confidence threshold of 70%.

The α-diversity was measured with the Ace, Chao, Shannon, Simpson and Coverage indexes using Mothur software. To identify differences in abundance in the gut microbiota between patients with NS and PS, the β-diversity was estimated by computing the Bray-Curtis (ANOSIM). Data are analyzed by wilcoxon rank-sum test with Benjamini-Hochberg false discovery rate multiple test correction. In the analysis of seeking significantly changed taxa between two groups, data were analyzed by wilcoxon rank-sum test with Benjamini-Hochberg false discovery rate multiple test correction. In LEfSe analysis, *p* < 0.05 (Kruskal–Wallis test) and log10[LDA] ≥ 3.0 were considered to indicate a significant difference in gut microbiota. *P* < 0.05 was considered statistically significant.

## Results

### Characteristics of participants

Based on the inclusion and exclusion criteria, fecal samples were collected from 35 patients with pathological scars and 40 patients with normal scars. In addition, the fecal samples derived from patients with pathological scars were distributed to the PS group and derived from patients with normal scars are distributed to the NS group. As seen in Table 1, there were no significant differences in gender, age, systolic blood pressure and diastolic blood pressure among the PS and NS groups. Details of the participants are shown in Table 1.

**TABLE 1:** Characteristics of participants.

	NS (*n* = 40)	PS (*n* = 35)	*P*-value
Gender, male/female	17/23	14/21	0.826
Age, years	28.92 ± 10.31	29.51 ± 11.12	0.812
BMI, kg/m^2^	21.75 ± 3.69	22.45 ± 3.07	0.378
Systolic blood pressure, mmHg	121.93 ± 8.78	119.20 ± 13.53	0.298
Diastolic blood pressure, mmHg	72.40 ± 6.69	71.31 ± 8.64	0.542
Blood glucose, mmol/L	4.67 ± 0.41	4.75 ± 0.56	0.508
Ca2+, mmol/L	2.33 ± 0.10	2.33 ± 0.12	0.987
Cholesterol, mmol/L	4.67 ± 0.89	4.44 ± 0.81	0.243
Dietary habits			
Meat (%)	13 (32.5)	11 (31.43)	0.921
Dessert (%)	11 (27.5)	12 (34.29)	0.618
Spicy food (%)	6 (15)	9 (25.71)	0.386

Data were compared using the χ^2^ test, or student *t*-test. *P* > 0.05 means no statistically significant difference.

### Alpha-diversity and beta-diversity analysis

**FIGURE 1:**
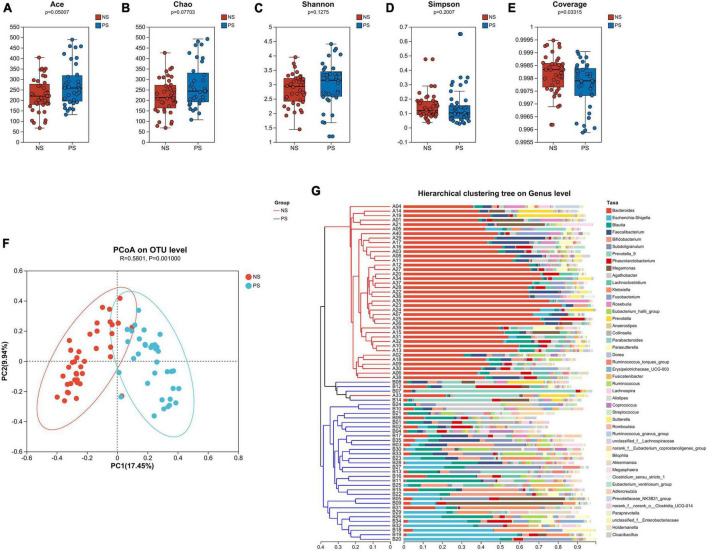
The α-diversity is analyzed by the **(A)** Ace, **(B)** Chao, **(C)** Shannon, **(D)** Simpson, **(E)** Coverage indexes. **(F)** The β-diversity is shown by principal coordinate analysis (PCoA) based on Bray–Curtis Dissimilarity index (ANOSIM, *R* = 0.5801, *P* = 0.0010). **(G)** A sample clustering tree and histogram structure analysis diagram at the genus level is shown. The hierarchical clustering analysis between samples based on community composition is on the left side and the right side indicate the histogram of a community composition of the samples.

Alpha-diversity and beta-diversity analysis had been performed to compare the similarities and differences in species diversity of the gut microbiota between the NS and PS groups. According to the α-diversity analysis, the Ace, Chao, Shannon and Simpson indexes indicated that PS group showed no significant difference of community diversity compared with the NS group (Figures 1A–D). However, Coverage index demonstrated that there was a significant difference between PS and NS group (Figure 1E).

The microbiota’s overall diversity was evaluated using the PCoA analysis based on the Bray-Curtis distance, and the results of the ANOSIM test indicated a significant difference between the PS and NS groups (*R* = 0.5801, *p* = 0.001, Figure 1F). The sample clustering tree and histogram combination analysis diagram (Figure 1G) clearly revealed that those with comparable β-diversity were grouped together, and the samples from the PS and NS groups could be well-clustered into two groups indicating a different composition of gut microbiota in PS and NS participants. Therefore, these findings revealed that PS patients exhibit dysbiosis, an imbalance in their gut microbial composition.

### Gut microbiome composition of PS and NS groups

In our study, Venn diagram showed that PS and NS groups shared 12 phyla, with 2 and 2 phyla unique to the NS group and PS group, respectively; shared 238 genera, with 20 and 58 genera unique to the NS group and PS group, respectively; shared 425 phyla, with 64 and 149 species unique to the NS group and PS group, respectively (Supplementary Figure 1).

**FIGURE 2:**
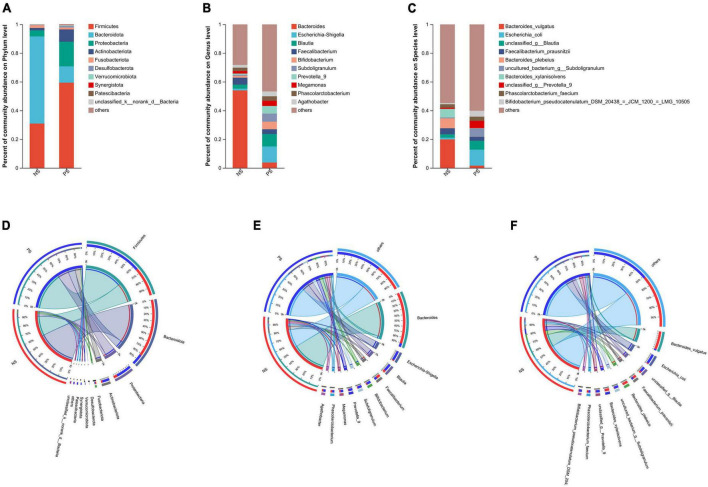
Relative abundance of microbial community at phylum, genus, species levels. **(A–C)** Bar plots show the average relative abundance of gut microbiota in NS, PS group. Circos analysis shows the corresponding abundance of fecal microbiota in NS, PS group at phylum **(D)**, genus **(E)**, species **(F)** levels.

Subsequently, as shown in Figure 2, we conducted bar plot and Circos analyses to illustrate the differences of the microbiota composition between PS and NS. The bar plot was used to roughly indicate the relative abundance of varied gut microbiota at phylum, genera and species levels (Figures 2A–C). At phylum level (Figure 2A), in the NS group, *Bacteroidota* was the most abundant phylum, followed by *Firmicutes, Proteobacteria and Fusobacteriota*, however, in the PS group, *Firmicutes* was the most abundant phylum, followed by *Proteobacteria, Bacteroidota, Actinobacteriota* and *Fusobacteriota*. The proportion of *Bacteroides* and *Faecalibacterium* was larger, while *Escherichia-Shigella*, *Blautia*, *Bifidobacterium* and *Subdoligranulum* were smaller in the NS group than the PS group at Genus level (Figure 2B). At species level (Figure 2C), NS patients were characterized by a higher relative abundance of *Bacteroides vulgatus* and a lower relative abundance of *Escherichia coli* compared with PS patients. The utilization of Circos analysis allowed for the depiction of the correlation in abundance of bacterial communities at the phylum (Figure 2D), genus (Figure 2E) and species (Figure 2F) levels between PS and NS, thereby corroborating the findings from the bar plot analysis.

### Comparison of the relative abundance of gut microbiota between PS and NS groups

**FIGURE 3:**
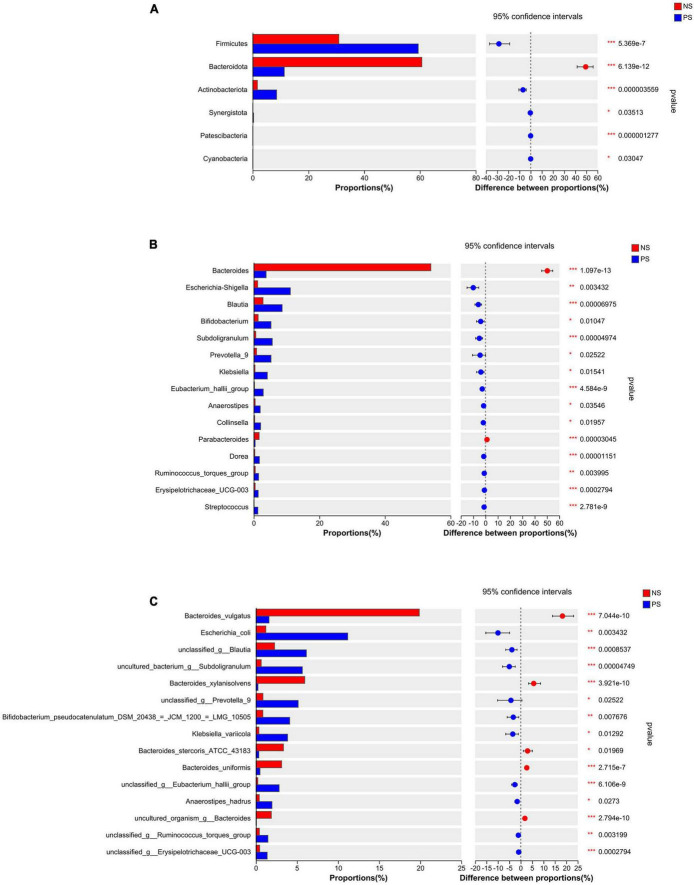
Comparison of the relative abundance of gut microbiota between NS and PS groups. Significantly changed taxa between two group at panel **(A)** phylum, **(B)** genus, **(C)** species levels. Data are analyzed by wilcoxon rank-sum test with Benjamini-Hochberg false discovery rate multiple test correction.

Those patients with PS displayed a relative difference of distinct gut microbiota, when compared with NS patients. The differentially abundant microbiota at the phylum, genus and species levels in PS versus NS were shown in Figure 3. At the phylum level (Figure 3A), PS showed a significant increase in *Firmicutes*, *Actinobacteriota*, *Synergistota*, *Patescibacteria* and *Cyanobacteria* but decrease in *Bacteroidota* compared with NS. At genus level (Figure 3B), the proportions of *Bacteroides* and *Parabacteroides* were significantly larger in NS, while *Escherichia-Shigella*, *Blautia, Bifidobacterium, Subdoligranulum, Prevotella, Klebsiella*, *Eubacterium hallii group, Anaerostipes, Collinsella, Dorea, Ruminococcus torques group, Erysipelotrichaceae UCG-003* and *Streptococcus* were significantly enriched in PS. At the species level (Figure 3C), NS showed a significant decrease in *Escherichia coli, unclassified Blautia, uncultured bacterium Subdoligranulum*, *unclassed Prevotella 9*, *Bifidobacterium pseudocatenulatum DSM 20438* = *JCM 1200* = *LMG 10505*, *Klebsiella variicola, unclassified Eubacterium hallii gourp*, *Anaerostipes hadrus, unclassified Ruminococcus torques group* and unclassified *Erysipelotrichaceae ucg-003*, while increase in *Bacteroides vulgatus*, *Bacteroides xylanisolvens*, *Bacteroides stercoris ATCC 43183*, *Bacteroides uniformis* and *uncultured organism Bacteroides*.

**FIGURE 4:**
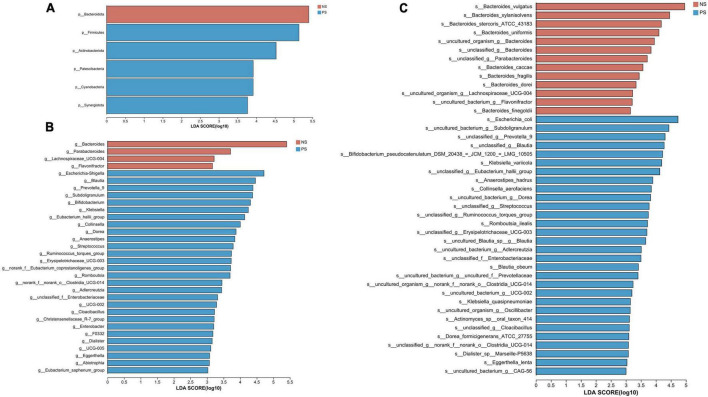
LDA diagram of LEfSe analysis at panel **(A)** phylum, **(B)** genus and **(C)** species levels. The red histogram represents NS group, and blue histogram represents PS group. The length of the histogram represents LDA score. *P* < 0.05 (Kruskal–Wallis test); log10[LDA] ≥ 3.0.

We conducted the LEfSe analysis to identify the biological taxonomic differences between PS and NS. We found 6, 31 and 43 differentially abundant taxa at the phylum level (Figure 4A), genus level (Figure 4B) and species level (Figure 4C), respectively. According to the LEfSe analysis, the PS group exhibited a predominance of *Firmicutes, Actinobacteriota*, and *Patescibacteria*, while presented a decrease in *Bacteroidota* at phylum level. At genus level, the proportion of *Escherichia-Shigella*, *Blautia, Prevotella 9* were larger, but the proportion of *Bacteroides, Parabacteroides* and Lachnospiraceae UCG-004 were smaller. At species level, PS showed a significant increase in *Escherichia coli*, *uncultured bacterium Subdoligranulum* and *unclassified Prevotella 9*, but decrease in *Bacteroides vulgatus*, *Bacteroides xylanisolvens and Bacteroides stercoris ATCC 43183*. More details of the differences were shown in Figure 4.

### Correlation network analysis

The gut microbiota forms an intricate network in which specific species are influenced not only by the host, but also by other bacteria present within the community. We performed a correlation network analysis to visualize the relationship between varied gut microbiota in PS and NS patients based on the top 50 relative abundance of OTUs. Among the 50 OTUs, 33 and 36 had associations with other OTUs in NS and PS respectively with an absolute coefficient value > 0.5, *p*-value < 0.05 (Supplementary Figure 2). Moreover, degree centrality (DC), closeness centrality (CC), and betweenness centrality (BC) centrality were performed to evaluate the taxa importance within the network (Supplementary Table 1).

**FIGURE 5:**
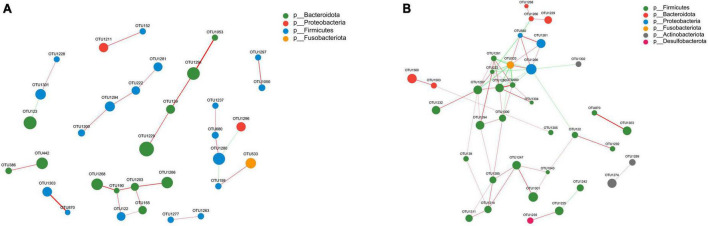
Correlation network analysis of the 50 most abundant OTUs for panel **(A)** NS and **(B)** PS. The networks display significant positive (red lines) and negative (green lines) correlations between operational taxonomic units (OTUs). The thickness of the lines represents the magnitude of the correlation coefficient, with thicker lines indicating a stronger correlation between OTUs. The size of the nodes represents the abundance of OTUs. OTUs are colored by phylum affiliation.

Notably, the correlation network of NS group (Figure 5A) showed a simpler and similar microbial relationship, however, the PS group (Figure 5B) presented a substantially more complicated microbial network. These findings suggested that gut microbial dysbiosis might exist in PS patients, and implied that the interaction changing between gut microbiota might led to the occurrence of pathological scars. In addition, in the gut microbial community network of PS patients, *Firmicutes* had a higher prevalence while *Bacteroidota* had a lower prevalence. Whereas, in the group of NS, both *Bacteroidota* and *Firmicutes* played dominant role in the correlation structure of gut microbiota. These results indicated that *Bacteroidota* and *Firmicutes* might play a crucial role in maintaining scar-related gut ecosystem in patients.

## Discussion

In recent years, there has been extensive research focused on the correlation between the human microbiome and various disorders. Currently, consistent evidence has demonstrated that the gut microbiota plays crucial roles in the development of skin diseases by interacting with the host system (Fang et al., 2021; Sinha et al., 2021; Moniaga et al., 2022). The current study focused on exploring the generalizable pathological scar-associated microbial signatures and determining the relationship between the gut microbiota and pathological scars using 16S rRNA gene sequencing technology with 35 PS patients and 40 NS patients’ fecal samples.

Regarding PS group, we found a significant difference in microbiota alpha diversity compared with NS group, and then we performed microbial beta-diversity analysis to examine the similarity in the overall community structure between two groups, and beta-diversity revealed a significant difference in microbiota community structure between PS and NS patients. The sample clustering tree and histogram combination analysis diagram obviously demonstrated that the samples from the PS and NS groups could be well-clustered into two groups, which indicated that there was a different composition of gut microbiota in PS and NS participants. Moreover, these results implied that gut microbial dysbiosis exists in PS patients.

There are four primary phyla in the human gut microbiota, which are named as *Actinobacteria*, *Bacteroidetes, Firmicutes*, and *Proteobacteria* (Arumugam et al., 2011). We found that the relative abundance of *Firmicutes* was higher in PS participants compared with NS group based on our 16s rRNA gene sequencing. In previous study, it was pointed out that the abundance of *Firmicutes* was increased in old female mice compared with the young ones with higher systemic inflammation (Ma et al., 2020). According to the prior research, we found that *Firmicutes* is closely associated with inflammatory diseases. During the preclinical stage of arthritis, the intestinal microbiota is primarily dominated by *Firmicutes* (Rogier et al., 2017). Hypertrophic scars and keloids are two types of pathological scars that result from differences in the intensity and duration of inflammation present in a wound, therefore, among them, hypertrophic scars are caused by mild inflammation, while keloids are caused by severe inflammation (Berman et al., 2017). In addition, Huang et al. (2013) claimed that the systemic factors in pathological scar patients could directly influence the process of angiogenesis, local inflammation, fibrosis and pathological scars remodeling. According to the prior research, we found that *Firmicutes* is closely associated with inflammatory diseases. Hence, these results implied that *Firmicutes* in human gut might play an important role in the occurrence of pathological scar via promoting systemic inflammatory response in the human.

At genus level, the proportion of *Bacteroides* was larger, while the ratio of *Escherichia-Shigella* was smaller in patients with NS. Previous study found that almost a quarter of the intestinal microbiota in humans is constituted by *Bacteroides*, making it a predominant genus (Ochoa-Repáraz et al., 2010). Due to their long-term existence in the host’s intestinal niche and co-evolution with the host, they have established a stable mutually beneficial symbiotic relationship (Faith et al., 2013). Since *Bacteroides* is significant decreasing in patients with inflammatory bowel disease (IBD) when compared with healthy control, it is considered to have potential anti-inflammatory properties (Takahashi et al., 2016; Brown et al., 2019; Zhong et al., 2019). In the opposite, *Escherichia-Shigella* is characterized by proinflammatory properties, prior studies revealed that the reduction of blood pro-inflammatory mediators may associated with the decreasing of *Escherichia-Shigella* in gut (Chen et al., 2021; Pivari et al., 2022). *Escherichia-Shigella* showed a positive correlation with inflammatory diseases in the human gut, however, *Bacteroides* demonstrated negatively correlated with inflammatory diseases, these results indicated that the adjustment of the proportion of *Bacteroides* and *Escherichia-Shigella* in the human gut may reshape the structure of the gut microbiota and subsequently improve the patient’s condition or prevent the occurrence or development of pathological scars.

On the changed species, we found that *Escherichia coli* significantly increased in PS group. An et al. (2021) demonstrated that the formation of CaOx stone in kidney can be facilitated by *Escherichia coli* through enhancing oxidative injury and inflammation. Moreover, it is found that *Escherichia coli* is responsible for the colorectal cancer susceptibility in patients by inducing inflammatory bowel disease (IBD) (Khan et al., 2017).

Franceschi et al. (2018) claimed that chronic and low-grade sterile inflammation throughout the body is an important pathogenic mechanism for various age-related diseases, including cardiovascular disease and metabolic diseases, they also demonstrate that gut microbiota plays a crucial role in both immunity and metabolism by constantly interacting with other organs and tissues throughout the body, resulting in significant effects. Pathological scars often occur after dermal injury and they are characterized by abnormal deposition of extracellular matrix and proliferation of fibroblasts (Lee et al., 2004; van der Veer et al., 2009; Huang and Ogawa, 2020). In clinical, a part of patients is prone to suffering from multiple pathological scars throughout the body for unknown reason. The delayed healing can be reversed through the use of exogenous estrogen treatment, applied either topically or systemically, via inflammation down-regulation inflammation (Ashcroft et al., 1997; Son et al., 2005). Moreover, xanthohumol and oxandrolone have been proven to accelerate wound healing by modulating the systemic inflammatory response (Costa et al., 2013; Ahmad et al., 2019). Therefore, the systemic inflammation caused by gut microbial dysbiosis may lead to the occurrence or development of pathological scars, it means that the microbiome directly or indirectly affects the balance of systemic inflammatory response in the human, and thus, perhaps, reshaping the proportion of microbiota in gut could improve the condition of patients who are susceptible to pathological scars.

Overall, based on the phylum, family, and genus levels, the current study found that the gut microbiota structure in patients who are susceptible to have pathological scars was different to that of patients with normal scars. Through their diverse biological effects, the varied gut microbiota played a crucial role on the occurrence and development of pathological scars by inducing systemic inflammation. Moreover, as shown in the correlation network, the interaction model between varied gut microbiota in patients with PS was greatly different to that of patients with normal scars.

## Conclusion

We provide a valuable and complete dataset, it will be helpful for the future studies which are aiming at exploring the relationship between gut microbiota and susceptibility to pathological scars. Our findings help clarify the proportion of gut microbiota in patients with PS and NS. In conclusion, our study has preliminary confirmed that dysbiosis exhibits in patients who are susceptible to pathological scars, and provide a new insight regarding the role of the gut microbiome in PS development and progression.

## Data availability statement

The datasets presented in the study are deposited in NCBI, accession number PRJNA980648.

## Ethics statement

The studies involving human participants were reviewed and approved by the Ethics Committee of the Fujian Medical University Union Hospital. Written informed consent to participate in this study was provided by the participants’ legal guardian/next of kin.

## Author contributions

XC, ZG, YX, and ML participated in the study design and provided the financial support. ML, YD, DL, YZ, HY, JLiu, HG, and JLin participated in the data acquisition. ML and MHL analyzed the data and wrote the manuscript. MHL, YD, and MH revised the manuscript. All authors read and approved the final version.

## Supplementary material

Supplementary Figure 1 OTU Venn diagram, red circle indicates the number of OTUs unique to NS, blue circle indicated the number of OTUs unique to PS, the overlap part shows the number of OTUs shared by the two groups.

Supplementary Figure 2 Heatmap of the 50 most abundant OTUs using Pearson correlation analysis. **(A)** NS group. **(B)** PS group. Correlation coefficient | r| ≥ 0.50, *P* < 0.05.

Supplementary Table 1 Network central coefficient table of NS and PS.

## References

[B1] Ahmad A. Herndon D. Szabo C. (2019). Oxandrolone protects against the development of multiorgan failure, modulates the systemic inflammatory response and promotes wound healing during burn injury. *Burns* 45 671–681. 10.1016/j.burns.2018.10.006 31018913

[B2] An L. Wu W. Li S. Lai Y. Chen D. He Z. (2021). *Escherichia coli* aggravates calcium oxalate stone formation via PPK1/flagellin-mediated renal oxidative injury and inflammation. *Oxidat. Med. Cell. Longev.* 2021:9949697. 10.1155/2021/9949697 34336124 PMC8292073

[B3] Arumugam M. Raes J. Pelletier E. Le Paslier D. Yamada T. Mende D. (2011). Enterotypes of the human gut microbiome. *Nature* 473 174–180. 21508958 10.1038/nature09944 PMC3728647

[B4] Ashcroft G. Dodsworth J. van Boxtel E. Tarnuzzer R. Horan M. Schultz G. (1997). Estrogen accelerates cutaneous wound healing associated with an increase in TGF-beta1 levels. *Nat. Med.* 3 1209–1215. 9359694 10.1038/nm1197-1209

[B5] Avram M. Tope W. Yu T. Szachowicz E. Nelson J. (2009). Hypertrophic scarring of the neck following ablative fractional carbon dioxide laser resurfacing. *Lasers Surg. Med.* 41 185–188. 10.1002/lsm.20755 19291746 PMC2747732

[B6] Berman B. Maderal A. Raphael B. (2017). Keloids and hypertrophic scars: Pathophysiology, classification, and treatment. *Dermatol. Surg.* 43 S3–S18. 27347634 10.1097/DSS.0000000000000819

[B7] Brown E. Ke X. Hitchcock D. Jeanfavre S. Avila-Pacheco J. Nakata T. (2019). *Bacteroides*-derived sphingolipids are critical for maintaining intestinal homeostasis and symbiosis. *Cell Host Microbe* 25 668–680.e7. 10.1016/j.chom.2019.04.002 31071294 PMC6544385

[B8] Chen S. Wu X. Yu Z. (2021). Juglone suppresses inflammation and oxidative stress in colitis mice. *Front. Immunol.* 12:674341. 10.3389/fimmu.2021.674341 34421890 PMC8375437

[B9] Chen S. Zhou Y. Chen Y. Gu J. (2018). Fastp: An ultra-fast all-in-one FASTQ preprocessor. *Bioinformatics* 34 i884–i890. 10.1093/bioinformatics/bty560 30423086 PMC6129281

[B10] Costa R. Negrão R. Valente I. Castela Â Duarte D. Guardão L. (2013). Xanthohumol modulates inflammation, oxidative stress, and angiogenesis in type 1 diabetic rat skin wound healing. *J. Nat. Prod.* 76 2047–2053. 10.1021/np4002898 24200239

[B11] D’Argenio V. Veneruso I. Gong C. Cecarini V. Bonfili L. Eleuteri A. (2022). Gut microbiome and mycobiome alterations in an in vivo model of Alzheimer’s disease. *Genes* 13:1564. 10.3390/genes13091564 36140732 PMC9498768

[B12] De Pessemier B. Grine L. Debaere M. Maes A. Paetzold B. Callewaert C. (2021). Gut-skin axis: Current knowledge of the interrelationship between microbial dysbiosis and skin conditions. *Microorganisms* 9:353. 10.3390/microorganisms9020353 33670115 PMC7916842

[B13] Faith J. Guruge J. Charbonneau M. Subramanian S. Seedorf H. Goodman A. (2013). The long-term stability of the human gut microbiota. *Science* 341:1237439. 10.1126/science.1237439 PMC3791589 23828941

[B14] Fang Z. Li L. Zhang H. Zhao J. Lu W. Chen W. (2021). Gut microbiota, probiotics, and their interactions in prevention and treatment of atopic dermatitis: A review. *Front. Immunol.* 12:720393. 10.3389/fimmu.2021.720393 34335634 PMC8317022

[B15] Fang Z. Pan T. Li L. Wang H. Zhu J. Zhang H. (2022). *Bifidobacterium longum* mediated tryptophan metabolism to improve atopic dermatitis via the gut-skin axis. *Gut Microb.* 14:2044723. 10.1080/19490976.2022.2044723 35239463 PMC8903757

[B16] Franceschi C. Garagnani P. Parini P. Giuliani C. Santoro A. (2018). Inflammaging: A new immune-metabolic viewpoint for age-related diseases. *Nat. Rev. Endocrinol.* 14 576–590. 10.1038/s41574-018-0059-4 30046148

[B17] Huang C. Akaishi S. Hyakusoku H. Ogawa R. (2014). Are keloid and hypertrophic scar different forms of the same disorder? A fibroproliferative skin disorder hypothesis based on keloid findings. *Int. Wound J.* 11 517–522. 10.1111/j.1742-481X.2012.01118.x 23173565 PMC7950391

[B18] Huang C. Murphy G. Akaishi S. Ogawa R. (2013). Keloids and hypertrophic scars: Update and future directions. *Plastic Reconstr. Surg. Glob. Open.* 1:e25. 10.1097/GOX.0b013e31829c4597 PMC4173836 25289219

[B19] Huang C. Ogawa R. (2020). The vascular involvement in soft tissue fibrosis-lessons learned from pathological scarring. *Int. J. Mol. Sci.* 21:2542. 10.3390/ijms21072542 32268503 PMC7177855

[B20] Jackson W. Nesti L. Tuan R. (2012). Mesenchymal stem cell therapy for attenuation of scar formation during wound healing. *Stem Cell Res. Ther.* 3:20. 10.1186/scrt111 PMC3392767 22668751

[B21] Jfri A. Rajeh N. Karkashan E. A. (2015). Case of multiple spontaneous keloid scars. *Case Rep. Dermatol.* 7 156–160. 26351423 10.1159/000437249 PMC4560309

[B22] Khan A. Khan Z. Malik A. Kalam M. Cash P. Ashraf M. (2017). Colorectal cancer-inflammatory bowel disease nexus and felony of *Escherichia coli*. *Life Sci.* 180 60–67. 10.1016/j.lfs.2017.05.016 28506682

[B23] Kim H. Lee D. Park S. Kim Y. Kim Y. Jeong J. (2014). Oral administration of *Lactobacillus plantarum* HY7714 protects hairless mouse against ultraviolet B-induced photoaging. *J. Microbiol. Biotechnol.* 24 1583–1591. 10.4014/jmb.1406.06038 25112318

[B24] Lau K. Paus R. Tiede S. Day P. Bayat A. (2009). Exploring the role of stem cells in cutaneous wound healing. *Exp. Dermatol.* 18 921–933. 19719838 10.1111/j.1600-0625.2009.00942.x

[B25] Lee J. Yang C. Chao S. Wong T. (2004). Histopathological differential diagnosis of keloid and hypertrophic scar. *Am. J. Dermatopathol.* 26 379–384. 15365369 10.1097/00000372-200410000-00006

[B26] Li J. Zhao F. Wang Y. Chen J. Tao J. Tian G. (2017). Gut microbiota dysbiosis contributes to the development of hypertension. *Microbiome* 5:14. 10.1186/s40168-016-0222-x 28143587 PMC5286796

[B27] Liu C. Zhao D. Ma W. Guo Y. Wang A. Wang Q. (2016). Denitrifying sulfide removal process on high-salinity wastewaters in the presence of *Halomonas* sp. *Appl. Microbiol. Biotechnol.* 100 1421–1426. 10.1007/s00253-015-7039-6 26454867

[B28] Lozupone C. Stombaugh J. Gordon J. Jansson J. Knight R. (2012). Diversity, stability and resilience of the human gut microbiota. *Nature* 489 220–230. 22972295 10.1038/nature11550 PMC3577372

[B29] Ma J. Hong Y. Zheng N. Xie G. Lyu Y. Gu Y. (2020). Gut microbiota remodeling reverses aging-associated inflammation and dysregulation of systemic bile acid homeostasis in mice sex-specifically. *Gut Microb.* 11 1450–1474. 10.1080/19490976.2020.1763770 32515683 PMC7524276

[B30] Magoč T. Salzberg S. L. (2011). FLASH: Fast length adjustment of short reads to improve genome assemblies. *Bioinformatics* 27 2957–2963. 10.1093/bioinformatics/btr507 21903629 PMC3198573

[B31] Mahmud M. Akter S. Tamanna S. Mazumder L. Esti I. Banerjee S. (2022). Impact of gut microbiome on skin health: Gut-skin axis observed through the lenses of therapeutics and skin diseases. *Gut Microb.* 14:2096995. 10.1080/19490976.2022.2096995 35866234 PMC9311318

[B32] Martin P. (1997). Wound healing–aiming for perfect skin regeneration. *Science* 276 75–81. 10.1126/science.276.5309.75 9082989

[B33] Moniaga C. Tominaga M. Takamori K. (2022). An altered skin and gut microbiota are involved in the modulation of itch in atopic dermatitis. *Cells* 11:3930. 10.3390/cells11233930 36497188 PMC9736894

[B34] Ochoa-Repáraz J. Mielcarz D. Wang Y. Begum-Haque S. Dasgupta S. Kasper D. (2010). A polysaccharide from the human commensal *Bacteroides fragilis* protects against CNS demyelinating disease. *Mucosal Immunol.* 3 487–495. 10.1038/mi.2010.29 20531465

[B35] Ogawa R. (2022). The most current algorithms for the treatment and prevention of hypertrophic scars and keloids: A 2020 update of the algorithms published 10 years ago. *Plastic Reconstr. Surg.* 149 79e–94e. 10.1097/PRS.0000000000008667 34813576 PMC8687618

[B36] Pai V. Cummings I. (2011). Are there any good treatments for keloid scarring after sternotomy? *Int. Cardiovasc. Thor. Surg.* 13 415–418. 10.1510/icvts.2010.264887 21737540

[B37] Pivari F. Mingione A. Piazzini G. Ceccarani C. Ottaviano E. Brasacchio C. (2022). Curcumin supplementation (Meriva(^®^)) modulates inflammation, lipid peroxidation and gut microbiota composition in chronic kidney disease. *Nutrients* 14:231. 10.3390/nu14010231 35011106 PMC8747135

[B38] Ramírez-Boscá A. Navarro-López V. Martínez-Andrés A. Such J. Francés R. Horga de la Parte J. (2015). Identification of bacterial DNA in the peripheral blood of patients with active psoriasis. *JAMA Dermatol.* 151 670–671. 10.1001/jamadermatol.2014.5585 25760018

[B39] Rogier R. Evans-Marin H. Manasson J. van der Kraan P. Walgreen B. Helsen M. (2017). Alteration of the intestinal microbiome characterizes preclinical inflammatory arthritis in mice and its modulation attenuates established arthritis. *Sci. Rep.* 7:15613. 10.1038/s41598-017-15802-x 29142301 PMC5688157

[B40] Salem I. Ramser A. Isham N. Ghannoum M. (2018). The gut microbiome as a major regulator of the gut-skin axis. *Front. Microbiol.* 9:1459. 10.3389/fmicb.2018.01459 30042740 PMC6048199

[B41] Sinha S. Lin G. Ferenczi K. (2021). The skin microbiome and the gut-skin axis. *Clin. Dermatol.* 39 829–839. 34785010 10.1016/j.clindermatol.2021.08.021

[B42] Son E. D. Lee J. Lee S. Kim M. Lee B. Chang I. (2005). Topical application of 17beta-estradiol increases extracellular matrix protein synthesis by stimulating tgf-Beta signaling in aged human skin in vivo. *J. Investig. Dermatol.* 124 1149–1161. 10.1111/j.0022-202X.2005.23736.x 15955089

[B43] Song H. Yoo Y. Hwang J. Na Y. Kim H. (2016). *Faecalibacterium prausnitzii* subspecies-level dysbiosis in the human gut microbiome underlying atopic dermatitis. *J. Allergy Clin. Immunol.* 137 852–860. 10.1016/j.jaci.2015.08.021 26431583

[B44] Takahashi K. Nishida A. Fujimoto T. Fujii M. Shioya M. Imaeda H. (2016). Reduced abundance of butyrate-producing bacteria species in the fecal microbial community in Crohn’s disease. *Digestion* 93 59–65. 26789999 10.1159/000441768

[B45] van der Veer W. Bloemen M. Ulrich M. Molema G. van Zuijlen P. Middelkoop E. (2009). Potential cellular and molecular causes of hypertrophic scar formation. *Burns* 35 15–29. 18952381 10.1016/j.burns.2008.06.020

[B46] Zheng D. Li R. An J. Xie T. Han Z. Xu R. (2020). Prebiotics-Encapsulated Probiotic Spores Regulate Gut Microbiota and Suppress Colon Cancer. *Adv. Mater.* 32:e2004529. 10.1002/adma.202004529 33006175

[B47] Zhong W. Lu X. Shi H. Zhao G. Song Y. Wang Y. (2019). Distinct microbial populations exist in the mucosa-associated microbiota of diarrhea predominant irritable bowel syndrome and ulcerative colitis. *J. Clin. Gastroenterol.* 53 660–672. 10.1097/MCG.0000000000000961 29210899

