# Supplementary figures and images for: 16S rRNA gene sequencing reveals the correlation between the gut microbiota and the susceptibility to pathological scars
Source: Front Microbiol. 2023 Jun 20;14:1215884. doi: 10.3389/fmicb.2023.1215884 (PMC10332274; doi:10.3389/fmicb.2023.1215884)

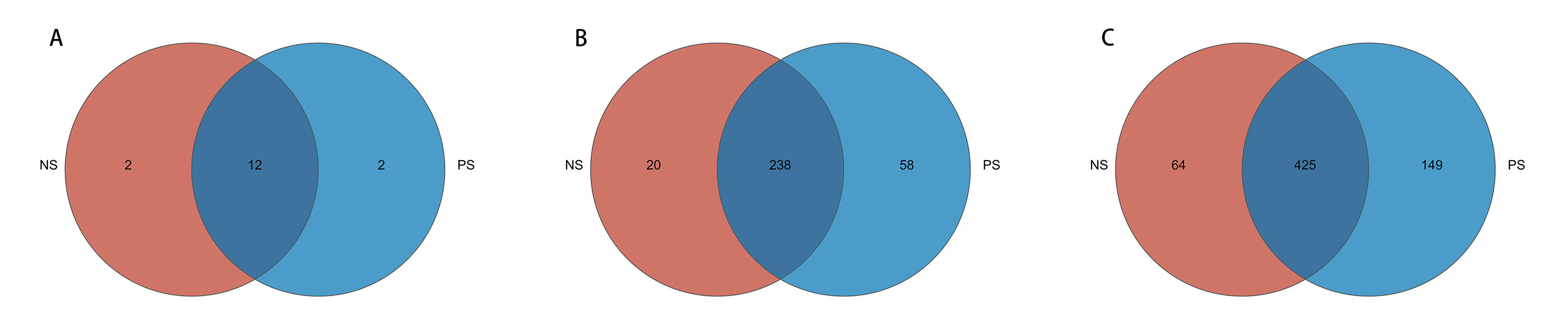

Supplement: Supplementary Figure 1 — OTU Venn diagram, red circle indicates the number of OTUs unique to NS, blue circle indicated the number of OTUs unique to PS, the overlap part shows the number of OTUs shared by the two groups. [file Image_1.JPEG]

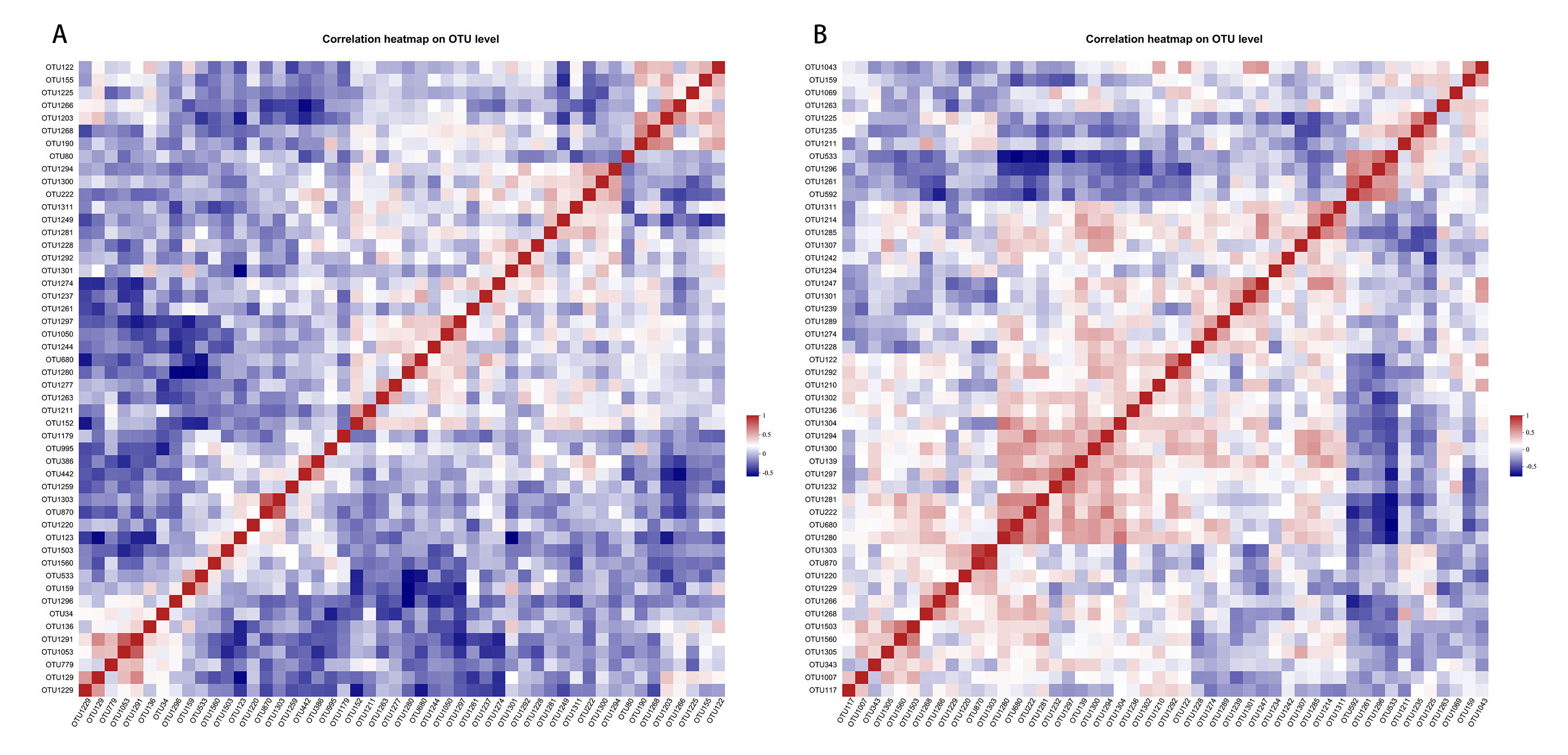

Supplement: Supplementary Figure 2 — Heatmap of the 50 most abundant OTUs using Pearson correlation analysis. (A) NS group. (B) PS group. Correlation coefficient | r| ≥ 0.50, P < 0.05. [file Image_2.JPEG]
