# Supplementary material for: 16S rRNA gene sequencing reveals the correlation between the gut microbiota and the susceptibility to pathological scars
Source: Front Microbiol. 2023 Jun 20;14:1215884. doi: 10.3389/fmicb.2023.1215884 (PMC10332274; doi:10.3389/fmicb.2023.1215884)
Supplement: Supplementary Table 1 — Network central coefficient table of NS and PS. [file Table_1.DOCX]

NS group

| Node ID | Node Name | Degree Centrality | Closeness Centrality | Betweenness Centrality |
| --- | --- | --- | --- | --- |
| 1 | OTU1229 | 0.03125 | 0.046875 | 0 |
| 2 | OTU129 | 0.0625 | 0.070313 | 0.004032 |
| 3 | OTU1291 | 0.0625 | 0.070313 | 0.004032 |
| 4 | OTU1053 | 0.03125 | 0.046875 | 0 |
| 5 | OTU1296 | 0.03125 | 0.071023 | 0 |
| 6 | OTU1280 | 0.09375 | 0.111607 | 0.016129 |
| 7 | OTU159 | 0.0625 | 0.086806 | 0.008065 |
| 8 | OTU533 | 0.03125 | 0.060096 | 0 |
| 9 | OTU123 | 0.03125 | 0.041667 | 0 |
| 10 | OTU1301 | 0.0625 | 0.0625 | 0.002016 |
| 11 | OTU870 | 0.03125 | 0.03125 | 0 |
| 12 | OTU1303 | 0.03125 | 0.03125 | 0 |
| 13 | OTU442 | 0.03125 | 0.03125 | 0 |
| 14 | OTU386 | 0.03125 | 0.03125 | 0 |
| 15 | OTU152 | 0.03125 | 0.03125 | 0 |
| 16 | OTU1211 | 0.03125 | 0.03125 | 0 |
| 17 | OTU1263 | 0.03125 | 0.03125 | 0 |
| 18 | OTU1277 | 0.03125 | 0.03125 | 0 |
| 19 | OTU680 | 0.0625 | 0.086806 | 0.008065 |
| 20 | OTU1237 | 0.03125 | 0.060096 | 0 |
| 21 | OTU1050 | 0.03125 | 0.03125 | 0 |
| 22 | OTU1297 | 0.03125 | 0.03125 | 0 |
| 23 | OTU1228 | 0.03125 | 0.041667 | 0 |
| 24 | OTU1281 | 0.03125 | 0.046875 | 0 |
| 25 | OTU222 | 0.0625 | 0.070313 | 0.004032 |
| 26 | OTU1294 | 0.0625 | 0.070313 | 0.004032 |
| 27 | OTU1300 | 0.03125 | 0.046875 | 0 |
| 28 | OTU190 | 0.09375 | 0.111607 | 0.010081 |
| 29 | OTU1268 | 0.03125 | 0.071023 | 0 |
| 30 | OTU1203 | 0.09375 | 0.111607 | 0.010081 |
| 31 | OTU122 | 0.0625 | 0.086806 | 0.002016 |
| 32 | OTU1266 | 0.03125 | 0.071023 | 0 |
| 33 | OTU155 | 0.0625 | 0.086806 | 0.002016 |

PS group

| Node ID | Node Name | Degree Centrality | Closeness Centrality | Betweenness Centrality |
| --- | --- | --- | --- | --- |
| 1 | OTU1305 | 0.028571 | 0.038095 | 0 |
| 2 | OTU1503 | 0.057143 | 0.057143 | 0.001681 |
| 3 | OTU1560 | 0.028571 | 0.038095 | 0 |
| 4 | OTU1268 | 0.028571 | 0.17337 | 0 |
| 5 | OTU1266 | 0.085714 | 0.22604 | 0.078992 |
| 6 | OTU1229 | 0.028571 | 0.17337 | 0 |
| 7 | OTU592 | 0.142857 | 0.302663 | 0.111821 |
| 8 | OTU870 | 0.028571 | 0.028571 | 0 |
| 9 | OTU1303 | 0.028571 | 0.028571 | 0 |
| 10 | OTU1280 | 0.228571 | 0.330688 | 0.031337 |
| 11 | OTU680 | 0.228571 | 0.330688 | 0.031337 |
| 12 | OTU222 | 0.228571 | 0.35014 | 0.056519 |
| 13 | OTU1281 | 0.171429 | 0.302663 | 0.004738 |
| 14 | OTU1297 | 0.228571 | 0.313283 | 0.08457 |
| 15 | OTU1300 | 0.142857 | 0.307882 | 0.053774 |
| 16 | OTU1304 | 0.057143 | 0.231911 | 0 |
| 17 | OTU1296 | 0.257143 | 0.343407 | 0.145274 |
| 18 | OTU533 | 0.257143 | 0.357143 | 0.067639 |
| 19 | OTU1294 | 0.114286 | 0.302663 | 0.031577 |
| 20 | OTU1232 | 0.028571 | 0.220459 | 0 |
| 21 | OTU139 | 0.057143 | 0.255102 | 0.021168 |
| 22 | OTU1214 | 0.114286 | 0.231911 | 0.019544 |
| 23 | OTU1285 | 0.142857 | 0.279018 | 0.077311 |
| 24 | OTU1302 | 0.028571 | 0.234962 | 0 |
| 25 | OTU1292 | 0.028571 | 0.202922 | 0 |
| 26 | OTU122 | 0.085714 | 0.279018 | 0.089252 |
| 27 | OTU1043 | 0.085714 | 0.241313 | 0.042033 |
| 28 | OTU1274 | 0.028571 | 0.028571 | 0 |
| 29 | OTU1289 | 0.028571 | 0.028571 | 0 |
| 30 | OTU1301 | 0.057143 | 0.200642 | 0 |
| 31 | OTU1247 | 0.114286 | 0.234962 | 0.039672 |
| 32 | OTU1242 | 0.028571 | 0.038095 | 0 |
| 33 | OTU1225 | 0.057143 | 0.057143 | 0.001681 |
| 34 | OTU1311 | 0.057143 | 0.207641 | 0 |
| 35 | OTU1261 | 0.085714 | 0.279018 | 0 |
| 36 | OTU1235 | 0.028571 | 0.038095 | 0 |
